# Refractory chronic migraine: a Consensus Statement on clinical definition from the European Headache Federation

**DOI:** 10.1186/1129-2377-15-47

**Published:** 2014-08-28

**Authors:** Paolo Martelletti, Zaza Katsarava, Christian Lampl, Delphine Magis, Lars Bendtsen, Andrea Negro, Michael Bjørn Russell, Dimos-Dimitrios D Mitsikostas, Rigmor Højland Jensen

**Affiliations:** 1 Department of Clinical and Molecular Medicine, Sapienza University of Rome, Rome, Italy; 2 Regional Referral Headache Centre, Sant’Andrea Hospital, Rome, Italy; 3 Department of Neurology, Evangelical Hospital, Unna, Germany; 4 Department of Neurology, University of Duisburg-Essen, Essen, Germany; 5 Headache Center Seilerstaette, Department of Neurogeriatric medicine and Remobilisiation, Hospital Barmherzige Schwestern Linz, Linz, Austria; 6 Department of Neurology, Headache Research Unit, University of Liège, Liège, Belgium; 7 Danish Headache Center, Department of Neurology, University of Copenhagen, Glostrup Hospital, Copenhagen, Denmark; 8 Head and Neck Research Group, Research Center, Akershus University Hospital, Lørenskog, Norway; 9 Institute of Clinical Medicine, Campus Akershus University Hospital, University of Oslo, Nordbyhagen, Norway; 10 Department of Neurology, Naval Hospital, Athens, Greece

**Keywords:** Chronic migraine, Refractory chronic migraine, Disease progression, rCM classification

## Abstract

The debate on the clinical definition of refractory Chronic Migraine (rCM) is still far to be concluded. The importance to create a clinical framing of these rCM patients resides in the complete disability they show, in the high risk of serious adverse events from acute and preventative drugs and in the uncontrolled application of therapeutic techniques not yet validated.

The European Headache Federation Expert Group on rCM presents hereby the updated definition criteria for this harmful subset of headache disorders. This attempt wants to be the first impulse towards the correct identification of these patients, the correct application of innovative therapeutic techniques and lastly aim to be acknowledged as clinical entity in the next definitive version of the International Classification of Headache Disorders 3 (ICHD-3 beta).

## Introduction

Migraine is the most frequent neurological disease observed in clinical practice. This primary headache is associated with an important socioeconomic impact [1,2] and the World Health Organization recognized the disorder as a major public health problem, by ranking it at 7th place among all worldwide diseases causing ictal disability [3,4].

Migraine is a paroxysmal disorder with a natural fluctuation between a low and a high frequency pattern in part influenced by modifiable and non-modifiable risk factors [5]. Increased attack frequency can lead to the so-called ‘chronic migraine’ (CM), which then becomes less responsive to acute as well as prophylactic migraine medications [6].

The understandable need to treat all the migraine attacks combined with a reduced efficacy of rescue medications, can determinate the occurrence of medication overuse [7].

All frequently used acute migraine medications, even when effective, seem to make the migraineurs brain more susceptible to migraine attack. In presence of CM and medication overuse, a vicious circle is built up, and the medication overuse becomes responsible of the persistence of the high frequency of the attacks (Medication Overuse Headache or MOH) and lack of responsiveness to the abortive and to most preventive medications.

The treatment of choice for those patients is the withdrawal of the overused drug either performed at home, using some advice and patient coaching, or in hospital settings exclusively for patients who failed ambulatory detoxification or seem to have a real addictive behavior [8-11]. This two-steps approach, education first and then hospitalization, seems to be the more real and reliable if we look at the 1 - 2% gross prevalence of MOH in the total population [12].

The response to a preventive drug varies from person to person and fluctuates over time. Moreover comorbidities like depression, insomnia, anxiety, hypertension and obesity act as worsening factors in the chronification process [13].

Despite substantial advances in migraine therapy some individuals with migraine are refractory to guideline-based treatment [14]. Additionally recent studies revealed that the majority of migraine patients are undertreated in terms of use of prophylactic drugs [15], thus favouring the progression of migraine into chronicity.

In these past years the need to offer a rescue treatment and a better prevention to the so-called medically intractable chronic headache patients has raised the possibilities for neuromodulation and met the interest of device producers willing to lend support to this complex clinical situation.

## The concept of refractory chronic migraine

The term CM is now well established in the clinical practice as well as in RCTs. The International Classification of Headache Disorders 3 beta (ICHD-3 beta) amended the main criteria of ICHD-2R for chronic migraine by adding the un-need to differentiate migraine with or without aura in the calculation of the monthly migraine days that must be 8 at least to the required criteria with a total of 15 headache days or more per month. Furthermore, the diagnosis can be ascertained even though a medication overuse exists, but in that case it is required that both CM and MOH diagnoses are added. The diagnosis of CM should then be revised after appropriate treatment of medication overuse as up to 3/4 of CM patients reverts to an episodic form after detoxification [8,10,11,16].

The term of refractory migraine has been used in the literature for a long time. In 1952 Reisman reported the first attempt to define refractory migraine by using a new experimental drug, namely ergot-alkaloids [17], but until recently, little attention has been paid to what it actually means to be refractory or how to define a patient as refractory.

Intractable migraine [18,19] is another term that has been used interchangeably for the headache types we are addressing. If we go through the semantic of these terms, it is easy to realize that they describe two different conditions. While a refractory headache can improve or worsen over time also in relation to events independent of the headache, an intractable headache carries in itself the implication that the condition may never be improved.

In our opinion the term “refractory” – which is more frequently used in the literature – should be preferred because it better emphasizes the lack of treatment response. Although the term refractory migraine has been used in the literature for decades, operational criteria were not defined until recently.

Table 1 shows the first attempt to systematize this controversial issue. The proposal of intractable headache in migraine introduced the concept of failure of at least four classes of preventative drugs for the first time [20]. Two years later, in 2008, the Refractory Headache Special Interest Section (RHSIS) of the American Headache Society (AHS) proposed the criteria for both refractory episodic migraine and refractory chronic migraine (rCM) (Table 1) [21]. According to this definition, rCM must fulfill the ICHD-2R criteria for CM [22], and headaches have to cause significant interference with function or quality of life despite modification of triggers, lifestyle factors, and adequate trials of acute and preventive medicines with established efficacy. This definition requires that patients with migraine fail adequate trials of preventive drugs, alone or in combination, from at least 2 of 4 drug classes including: beta-blockers, anticonvulsants, tricyclics, and calcium channel blockers, whereas the term adequate is not further specified. Patients must also fail adequate trials of abortive medicines, including both a triptan and dihydroergotamine (DHE) intranasal or injectable formulation and either nonsteroidal anti-inflammatory drugs (NSAIDs) or combination analgesic, unless contraindicated. Since the RHSIS criteria, other proposed definitions included a rating scale to delineate the degree of intractability [23] and defined certain issues of treatment failure more precisely [24]. One aspect before considering a CM patient “refractory” to preventive therapy was the maximum possible number of drugs that had to be tested and found ineffective (Table 1) [25]. Likewise the beneficial use of multidisciplinary team in these difficult to treat patients is not further specified or requested despite guidelines recommendations.

**Table 1:** Previous clinical definition of refractory chronic migraine

**Intractable headache (Goadsby (2006)**	**RHSIS criteria (AHS 2008)**	**Refractory Migraine (after D’Amico 2008)**
Failed an adequate trial of regulatory approved and conventional treatments according to local national guidelines	A. ICHD-II migraine or chronic migraine	CM patients for whom adequate trials of preventive therapies at adequate doses have failed to reduce headache frequency and improve headache-related disability.
In migraine, failure of at least 4 classes, where 3 should come from 1 to 4	B. Headaches cause significant interference with function or quality of life despite modification of triggers, lifestyle factors, and adequate trials of acute and preventive medicines with established efficacy	MOH patients should also be considered refractory when treatments fail to reduce the consumption of symptomatic drugs.
1. Beta-blockers	1. Failed adequate trials of preventive medicines, alone or in combination, from at least 2 of 4 drug classes:	*Preventive drugs*
2. Anticonvulsants	a. Beta blockers	The greatest possible number of drugs should be tested and found ineffective (or intolerable).
3. Calcium channel blockers	b. Anticonvulsants	It is not sufficient to try one medication of each pharmacological class.
4. Tricylic antidepressants	c. Tricyclics	*Adequate trial*
5. Other treatments with at least 1 positive randomized controlled trial	d. Calcium channel blockers	Adequate courses of all drugs considered as first-line prophylactics for episodic migraine by international guidelines, and in addition adequate courses of at least some of the drugs considered second- or third-line prophylactic treatments.
6. Nonsteroidal anti-inflammatory drugs	2. Failed adequate trials of abortive medicines from the following classes, unless contraindicated:	*Trial duration and dosage*
7. Metabolic enhancers, such as vitamin B2 or coenzyme Q10	• Both a triptan and DHE intranasal or injectable formulation	A 3-month treatment period is required to assess efficacy but it may be useful to continue for a further 3–6 months if there was some improvement during the first 3 months.
*Adequate trial*	• Either non-steroidal anti-inflammatory drugs or combination analgesics	*Treatment of medication overuse*
Appropriate dose	*Adequate trial*	Acute medication overuse should be curtailed before starting prophylaxis in patients with chronic headaches.
Appropriate length of time	Period of time during which an appropriate dose of medicine is administered, typically at least 2 months at optimal or maximum-tolerated dose, unless terminated early due to adverse effects	*Treatment of comorbidities*
Consideration of medication overuse	*Modifiers*	Identification and appropriate treatment of all clinically significant comorbidities is essential before declaring a treatment failure in CM patients.
*Failed*	1. With or without medication overuse, as defined by ICHD-2	
No therapeutic or unsatisfactory effect	2. With significant disability, as defined by MIDAS ≥11	
Intolerable side effects		
Contraindications to use		

Some authors may argue that it wouldn’t be enough to try one medication of each pharmacological class (e.g., one beta-blocker, one anticonvulsants, etc.) as the members of a given class may work by various mechanisms and a patient unresponsive to one molecule may improve with another, and tolerability within a class varies too [25]. However, since the 2008 RHSIS proposal something has changed in the treatment scenario for chronic migraine patients. The results from the PREEMPT studies, published in 2010, have shown the efficacy and safety of onabotulinumtoxinA for the preventive treatment of CM [26] and it should also be added to the list of preventive therapy to try before labeling a migraine patient as refractory.

Despite the definitions provided by the current ICHD-3 beta it does not include a definition of refractoriness in migraine [27]. A growing need of a shared definition of refractoriness has already been claimed from a multidisciplinary expert group [28].

It is not surprising that so far no consensus regarding the definition of rCM has emerged. It is still being debated what should be the key parameter of a definition of refractoriness (e.g., unresponsiveness to treatment, high frequency, severe disability or all of these features) and if refractory headache should be considered as a single entity or rather a hard treatable version of different headache disorders [23].

Our opinion is that the definition of rCM should be based on the non-responsiveness to preventative treatment, not on the non-responsiveness to acute treatment. In fact the key for success is prevention: refractoriness is the consequence of prophylaxis failure while medication overuse headache can be both the cause and the consequence of the refractoriness itself. Therefore it is required that medication overuse headache should be ruled out or be adequately treated before a patient can be classified as refractory.

The attempt to define rCM has to keep in consideration what are meant to be the operational purposes of that classification as RCTs, referral from a primary care provider to a headache specialist, medical cost reimbursement, screening tool for invasive treatment or implantable devices. With the need of minimizing the risk of a misdiagnosis we have to exclude the possible causes of a false refractoriness and to focus on the small group of truly refractory patients.

A special attention should be paid to the very frequent presence of comorbidities (psychiatric and/or somatic) in this subset of rCM patients. Depression and anxiety disorders represent undisputable co-factors in the progression of migraine chronification and must be adequately treated [29-32].

Preventive medication should be preferably used as monotherapy, since our knowledge of combining different preventive medications is sparse. The combination topiramate and propranolol did not have any synergy effect and was not superior to either preventive medication given in monotherapy [33]. On the other hand, additional treatment of comorbidities is needed, either pharmacological or psychological, or even better a combination with a multidisciplinary team when available.

Using the criteria proposed by RHSIS 5.1% of the migraine patients evaluated in an US- headache clinic is diagnosed as refractory [21]. However until a well-accepted definition is formulated evidence-based treatment recommendations for rCM cannot be generated.

The clinical complexity of rCM moved the scientific interest to new concepts by studying interesting but still not sufficiently validated approaches, e.g. neuromodulation [28,34].

The European Headache Federation (EHF) felt the need to develop new consensus criteria that define rCM, particularly for the purposes of controlled clinical trials that involve experimental medication and neuromodulation independently of the non-invasive therapies or the implantable devices.

Considering rCM as an evolution of CM, we can hypothesize the inclusion of rCM as a 3-digit diagnosis of CM (1.3.1 Refractory chronic migraine) (see Table 2).

**Table 2:** European Headache Federation proposed criteria for refractory chronic migraine

**EHF proposed criteria for refractory chronic migraine**	
A. ICHD-III β chronic migraine	
No medication overuse	
B. Prophylactic migraine medications in adequate dosages used for at least 3 months each.	
C. Contraindications or No effect of the following preventive medication with at least 3 drugs from the following classes:	
• Beta blockers	
propranolol up to 240 mg/d	
metoprolol up to200mg	
atenolol up to100mg	
bisoprolol up to10mg	
• Anticonvulsants	
Valproate acid up to 1,5 g/d	
Topiramate up to 200 mg/d	
• Tricyclics	
amytriptyline up to 150 mg/d	
• Others	
Flunarizine up to 10 mg/d	
Cardesartan 16 mg/d	
• OnabotulinumtoxinA	
155 - 195 U according to the PREEMPT protocol	
D. Adequate treatment of psychiatric or other comorbidities by multidisciplinary team, if available.	
*Notes:*	
*-* Secondary Headache must be excluded	
- MRI provides no underlying cause	
- Laboratory and CSF analyses within normal range, including CSF pressure	
- Meaning of efficacy: reduction on HA days >50%	
- Detoxification procedure (in/out hospital setting): intravenous, oral and advice only are all accepted.	

## Conclusions

It is our opinion that exclusively headache experts should conduct the management of this migraine population particularly difficult to treat.

This EHF definition of rCM has to be considered as a mandatory tool in any multidisciplinary or innovative therapeutic approach.

The principal task of this EHF Expert Group Consensus Statement is to bring the definition of rCM up to date. For too long there has been a lot of utterance about it while their nosography was not systemized. So far few innovative neuromodulation practices have been widely applied to this subset of headaches, numerically limited but with a severe impact in terms of disability and social costs. Therapeutic results are before our eyes, still too scanty and often with weak scientific prerequisites. The lack of necessary evidence and its validation has made possible that, in the recent ICHD-3 β, refractoriness has found no room. It would be very valuable to scotomize this subset of headache patients with clear universal definitions instead of entrusting them only to striking case series without a scientific definition of refractoriness. This issue too must be investigated further in the course of the explorative work on refractoriness of headaches and its boundaries, by carefully field testings and using updated clinical criteria for rCM.

## Competing interest

PM received travel grants, consulting fees or unrestricted grants from Nevro Corporation, St Jude Medical, Allergan, Pfizer, ACRAF, is member of Advisory Board in Allergan and St Jude Medical as well as director in LTB and EHF.

RHJ has given lectures for Pfizer, Berlin-Chemie, Allergan, Merck, ATI. Is also member of advisory boards in: ATI, Medotech, Neurocore, and Linde Gas as well as director in LTB, EHMTIC and President in EHF.

CL serves on scientific advisory boards for Allergan, Bayer HealthCare and St. Jude Medical; has received funding for travel from Bayer Schering Pharma, Pfizer, Allergan; served as a consultant to Bayer Schering Pharma, Biogen Idec; received research support from Bayer Schering Pharma, Allergan, Biogen Idec; has received personal compensation for consultations or lectures from Bayer HealthCare, Sanofi Aventis, Biogen Idec, Teva Pharmaceuticals, Pfizer.

DDM is member of advisory boards in Allergan, Astellas, Bayer-Schering, Novartis, Genzyme-Sanofi, Merck-Serono, Genesis Pharma, Teva, and has received honoraria for lecturing from Pfizer, Lilly, Menarini and UCB.

DM has received travel grants from Allergan and research funds from Neurocore.

ZK, AN, DM and MBR declared no competing interests related to the contents of this Consensus Statement.

In details they had not received any research funds, travel or unrestricted grants, consulting fees, honoraria as speaker or consultant from the drug companies of the medicines or devices mentioned above.

Furthermore, all authors declare to not own any stock option of the manufacturers of drugs discussed in this review.

Finally, all the authors state they have received no direct or indirect payment in preparation of this manuscript.

## Authors’ contributions

All Authors on behalf of European Headache Federation contributed equally to the conception, design, drafting and critical revisions of the manuscript. The final version has been approved by all Authors.
